# CARARIME: Interactive web server for comprehensive analysis of renal allograft rejection in immune microenvironment

**DOI:** 10.3389/fimmu.2022.1026280

**Published:** 2022-11-17

**Authors:** Xiaoyou Liu, Ding Liu, Song Zhou, Weihao Jiang, Jie Zhang, Jianmin Hu, Guorong Liao, Jun Liao, Zefeng Guo, Yuzhu Li, Siqiang Yang, Shichao Li, Hua Chen, Ying Guo, Min Li, Lipei Fan, Liuyang Li, Ming Zhao, Yongguang Liu

**Affiliations:** ^1^ Department of Organ Transplantation, The First Affiliated Hospital of Guangzhou Medical University, Guangzhou, China; ^2^ Department of Organ Transplantation, Zhujiang Hospital of The Southern Medical University, Guangzhou, Guangdong, China

**Keywords:** CARARIME, immune microenvironment, renal allograft rejection, web server, kidney allograft rerejction

## Abstract

**Background:**

Renal transplantation is a very effective treatment for renal failure patients following kidney transplant. However, the clinical benefit is restricted by the high incidence of organ rejection. Therefore, there exists a wealth of literature regarding the mechanism of renal transplant rejection, including a large library of expression data. In recent years, research has shown the immune microenvironment to play an important role in renal transplant rejection. Nephrology web analysis tools currently exist to address chronic nephropathy, renal tumors and children’s kidneys, but no such tool exists that analyses the impact of immune microenvironment in renal transplantation rejection.

**Methods:**

To fill this gap, we have developed a web page analysis tool called Comprehensive Analysis of Renal Allograft Rerejction in Immune Microenvironment (CARARIME).

**Results:**

CARARIME analyzes the gene expression and immune microenvironment of published renal transplant rejection cohorts, including differential analysis (gene expression and immune cells), prognosis analysis (logistics regression, Univariable Cox Regression and Kaplan Meier), correlation analysis, enrichment analysis (GSEA and ssGSEA), and ROC analysis.

**Conclusions:**

Using this tool, researchers can easily analyze the immune microenvironment in the context of renal transplant rejection by clicking on the available options, helping to further the development of approaches to renal transplant rejection in the immune microenvironment field. CARARIME can be found in http://www.cararime.com.

## Introduction

Renal failure is a serious disease which can cause fatal complications if patients are not placed on hemodialysis in time, to remove waste and excess fluid from the blood (1). Kidney transplant is one treatment approach for end-stage renal disease that can significantly improve the survival rate and quality of life. However, clinical application is restricted by the high rate of immune rejection. Recent studies have shown that the incidence of acute rejection is ~20% and of chronic rejection ~40%. Therefore, it is of great significance to further study the specific mechanisms leading to renal transplant rejection.

It has been shown that the complex interaction between the immune microenvironment and kidneys is an important factor in the rejection of renal transplants, with T cells, donor anti-presenting cells (APCs), and natural killer cells (NKs) all playing a vital role. Patients’ macrophages recognize and process the donor’s allogeneic antigen, which is then presented to T cells for recognition, finally forming an interstitial inflammatory zone (2, 3). Additionally, CD8+T and cytotoxic T lymphocytes (CTLs) can directly attack inhibitors through cytotoxic effects (e.g., IFN-γ, granzyme, and perforin) and by directly homing to the graft, causing rejection. In addition, T helper 17 (Th17) cells have been shown to play an important role in the occurrence of acute and chronic rejection in animal models of allogeneic transplantation (4, 5), and that this effector Th17 cell is mainly regulated by Treg cells (6). It follows that an insufficiency in Treg cell number or function is closely related to the occurrence of organ rejection (7). Besides immune cells, inflammatory mediators such as tumor necrosis factor TNF-α (8), chemokine MCP-1, and macrophage inflammatory protein (MIP)-1 play an important role in the occurrence and development of chronic rejection, by promoting tissue inflammation (9).

Recent studies that use high-throughput sequencing to compare rejection patients after renal transplantation with non-rejection or rejection patients has produced considerable RNA-seq data, providing an unprecedented opportunity to explore the immune microenvironment characteristics of these patients (10–16). For example, Reeve et al. collected and analyzed 1,745 samples of renal allograft from rejection and non-rejection patients after renal transplantation (10). Friedewald and his colleagues simultaneously detected the expression data from 530 blood samples by microarray, comparing a rejection group and control group after kidney transplantation (12). Halloran et al. sequenced 294 rejected and normal kidney tissues after kidney transplantation, and Zhang and his colleagues performed high-throughput sequencing (RNA-Seq) on blood samples of 235 patients after kidney transplantation. In order to more comprehensively collect the data of renal transplant rejection samples published in the Gene Expression Omnibus (GEO) database, we included a total of 6,178 renal transplant rejection and control samples *via* manual retrieval and validation.

Currently, the online database and analysis tools related to nephrology are extremely limited (17–19): Xu et al. has developed a web tool to predict the progression of chronic kidney disease (17) (https://ncutool.shinyapps.io/CKDprogression/), Maria has developed a web tool that can evaluate the length and volume of children’s kidneys (18), and Xi et al. have developed a web tool called OSkirc that can be used to screen the prognostic markers of renal clear cell carcinoma. In general, most medical web analysis tools [such as Xena (20), CBioportal (21) [http://www.cbioportal.org/], Human Protein Atlas portal [HPA] (22), Expression Atlas (23), GEPIA (24), and GEPIA2 (25)] are based on oncology. We find that there is a great lack of comprehensive analysis web tools for renal transplant rejection cohorts and that the current web tools do not provide follow-up analysis of the immune microenvironment specific to renal transplant rejection samples, such as immune cell analysis (difference analysis, correlation analysis), signal pathway enrichment analysis (GSEA, ssGSEA), and analysis of the influence of immune cells and signal pathways on renal transplant rejection (ROC, logistics regression, univariable-Cox regression, Kaplan-Meier). Based on the above unmet needs, we developed a web-based tool that provides fast and customizable functions to supplement the existing tools: Comprehensive Analysis of Renal Allograft Rerejction in Immune Microenvironment (CARARIME). CARARIME can be found in http://www.cararime.com.

## Methods

### Data collection

Graft rejection and normal samples after renal transplantation were downloaded from the GEO database, and data sets with no less than 10 samples were included. For data obtained by chromatin immunoprecipitation (ChIP) test, expression values were normalized by the normalizeBetweenArrays function in the Limma package. For RNA-seq data, fragments per kilobase of exon model per million mapped fragments (FPKM) was used for subsequent analysis. For details of all samples in CARARIME, see **Table S1**.

### CARARIME web application

**Figure 1:**
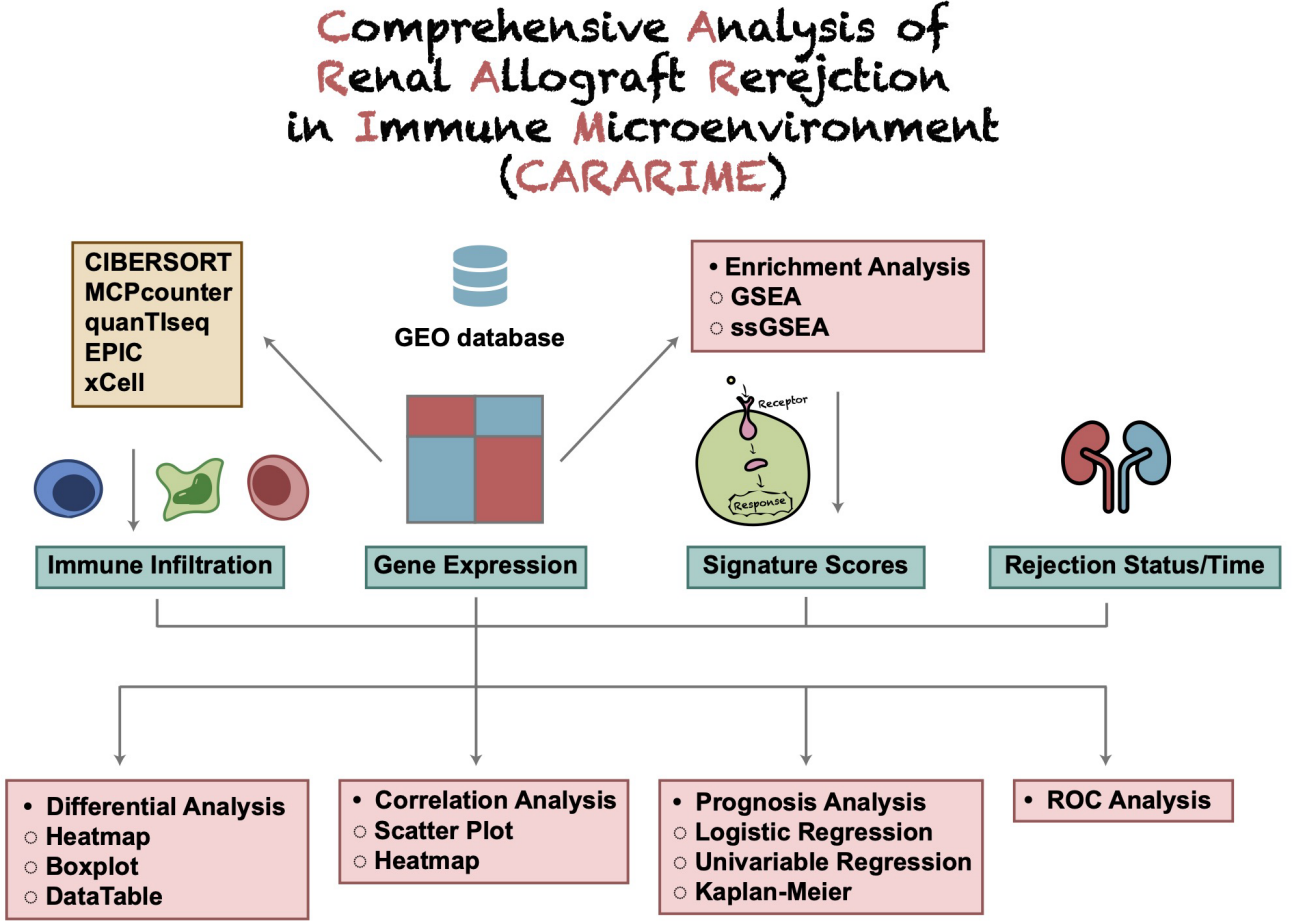
The flowchart of CARARIME construction.

The CARARIME web tool is based on the Shiny package (ui and sever) and HyperText Markup Language (HTML) and does not require users to register an account. The immune score expression data were evaluated by the Cibersort, xCell, MCPcounter, EPIC, and quanTIseq algorithms. For difference analysis, the limma package was used to analyze the rejection and control groups. Kaplan-Meier and Univariable-Cox regression analysis was carried out using the survival and survminer packages. Heat maps were visualized by the ComplexHeatmap package, box graphs and scatter graphs were visualized using the ggpubr package, and the table DT package was used to present the data. The stats package was used for logistics regression analysis, and the clusterProfiler R package used for GSEA analysis. The forest map was drawn using the ggplot2 package and ROC curve drawn using the pROC package. Users can download data using the “Download CSV” option. In addition, users can use the “Download PDF” option to save the analyzed figure. An overview of the CARARIME workflow is shown in **Figure 1**.

The interface of CARARIME is divided into seven main tabs: differential analysis, prognosis analysis, correction analysis, enrichment analysis, ROC analysis, dataset, and frequently asked questions (FAQ). We merged the three datasets (GSE48581, GSE98320, and GSE124203) into one dataset and randomly divided this dataset into a training dataset and internal validation dataset (6:4). The immune cells scores of the training dataset and internal validation dataset were used to predict the renal transplant rejection status of the kidney transplant recipients (KTRs) based on the univariable logistic regression model. We used the multivariable logistic regression model and 10-fold cross-validation to construct a weighted risk score model based on the values of the selected predictors using the logistic regression coefficients from the training set. We used GSE25902, GSE129166, GSE36059, GSE131179, GSE72925, and GSE47097 as the external validation set, and calculated the risk scores of each sample. The receiver operating characteristic (ROC) curve was used to verify the effectiveness of the risk score model.

## Results

### Home

The CARARIME home page provides a simple text introduction and the analysis functions of the web tool. In addition, users can view all the analysis results provided by CARARIME *via* clicking the slide buttons on the left and right sides of the picture. For the CARARIME database, we collected a total of 29 cohorts related to renal transplantation rejection, including 6,178 samples. Further details about the data sets included in CARARIME can be found in **Table S1**.

### Differential analysis

**Figure 2:**
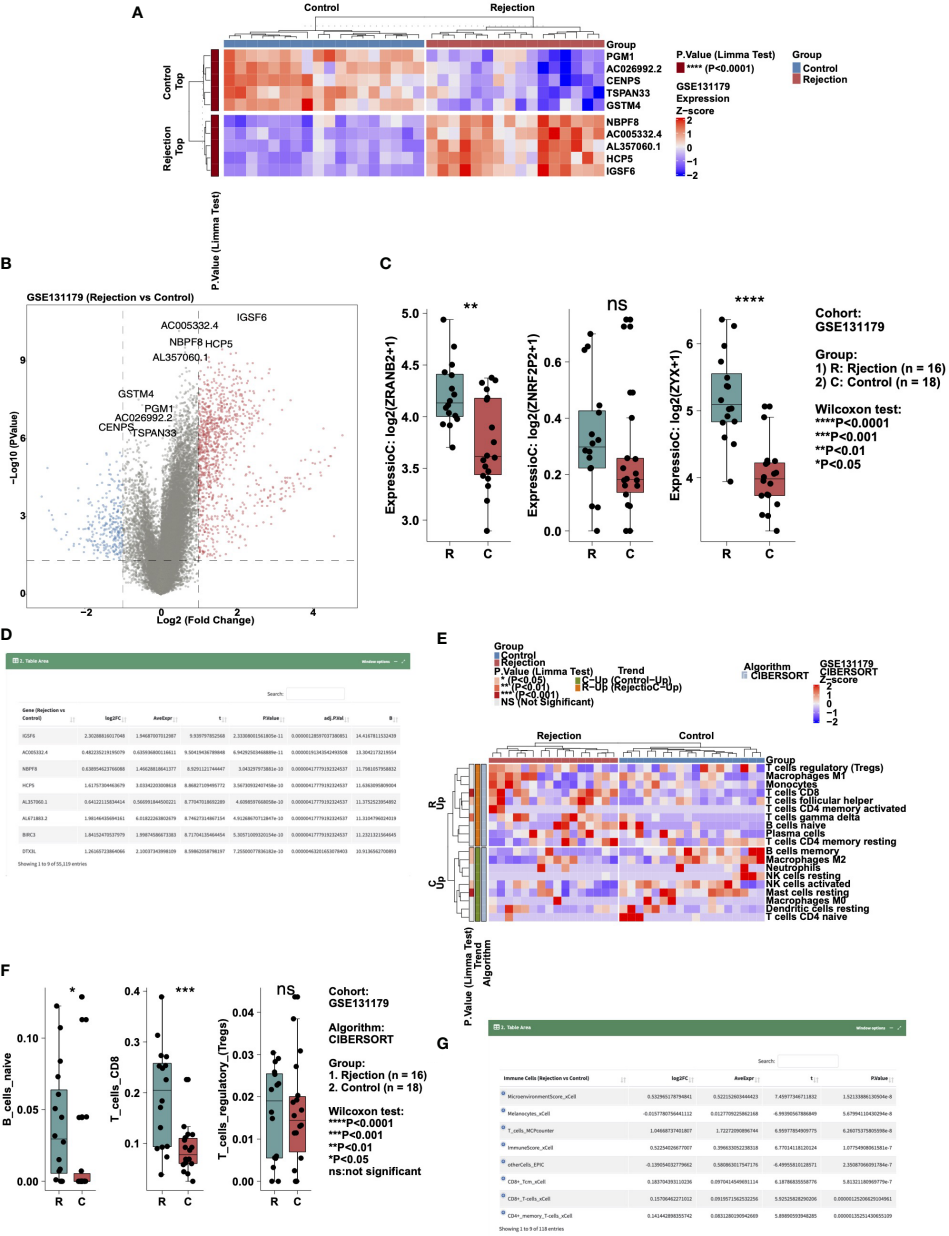
Example of the “Differential Analysis” tab. **(A)** Heatmap showing the top5 and down5 gene expression between the rejection and control group in cohort GSE131179. **(B)** Volcano plot showing the differential expression of genes between the rejection and control group in cohort GSE131179. **(C)** Boxplot showing the differences in the gene expressions of ZRANB2, ZNRF2P2, and ZYX between the rejection and control group in cohort GSE131179. **(D)** Data table showing the result of the differential expressing genes analysis. **(E)** Heatmap showing the immune cell scores estimated by CIBERSORT in the GSE131179 cohort. **(F)** Boxplot showing the differences in the score of B_cells_naive, T_cells_CD8, and T_cells_regulatory_(Tregs) between the rejection and control group in cohort GSE131179. **(G)** Data table showing the result of the differential immune cell scores between the rejection and control group in cohort GSE131179.

To analyze the difference in expression data in a certain cohort, users can select the “Gene Expressions” sub-tab. In “Heatmap,” users can, for example, select “Cohort: GSE131179; Top/Down-Regulated Gene: 5; Cluster Method: ward.D” to show the expression levels of five genes with different expression trends (up- and down-regulation) in the rejection and control groups in the form of a heat map (**Figure 2A**). In “Volcano Plot,” users can show the differentially expressed genes that were significantly up- and down- regulated according to the above cut-off in the rejection and control groups (Red: significantly up-regulated; Blue: significantly reduced; Gray: no significant change). An example using the parameters “Cohort: GSE131179; P value: 0.05; |Log2FC|:1” is shown in **Figure 2B**. In “Boxplot,” users can, for example, select “Cohort: GSE131179; Gene symbol (max: 10): ZRANB2, ZNRF2P2, ZYX” to display the results of a Wilcox test conducted in the rejection and control groups on the expression of the above genes (**Figure 2C**). In “DataTable,” users can choose to analyze the difference analysis results of gene expression between different cohort samples (such as GSE131179, shown in **Figure 2D**).

To analyze the difference in immune cell scores in a cohort, users can select the “Immune Cells” sub-tab. In “Heatmap,” users can, for example, select “Cohort: GSE131179; Algorithm: CIBERSORT; Cluster Method: ward.D” to show the immune cells with different expression trends (up- and down-regulation) in the rejection and control groups in the form of a heat map (**Figure 2E**). In “Boxplot,” users can, for example, select “Cohort: GSE131179; Algorithm: CIBERSORT; Immune Cells (max:10): B_cells_naive, T_cells_CD8, T_cells_regulatory_(Tregs)” to perform a Wilcox test in the rejection and control groups for the aforementioned immune cell scores (**Figure 2F**). In “DataTable,” users can choose to analyze the difference analysis results of immune cell scores in different cohort samples (such as GSE131179, shown in **Figure 2G**).

### Prognosis analysis

**Figure 3:**
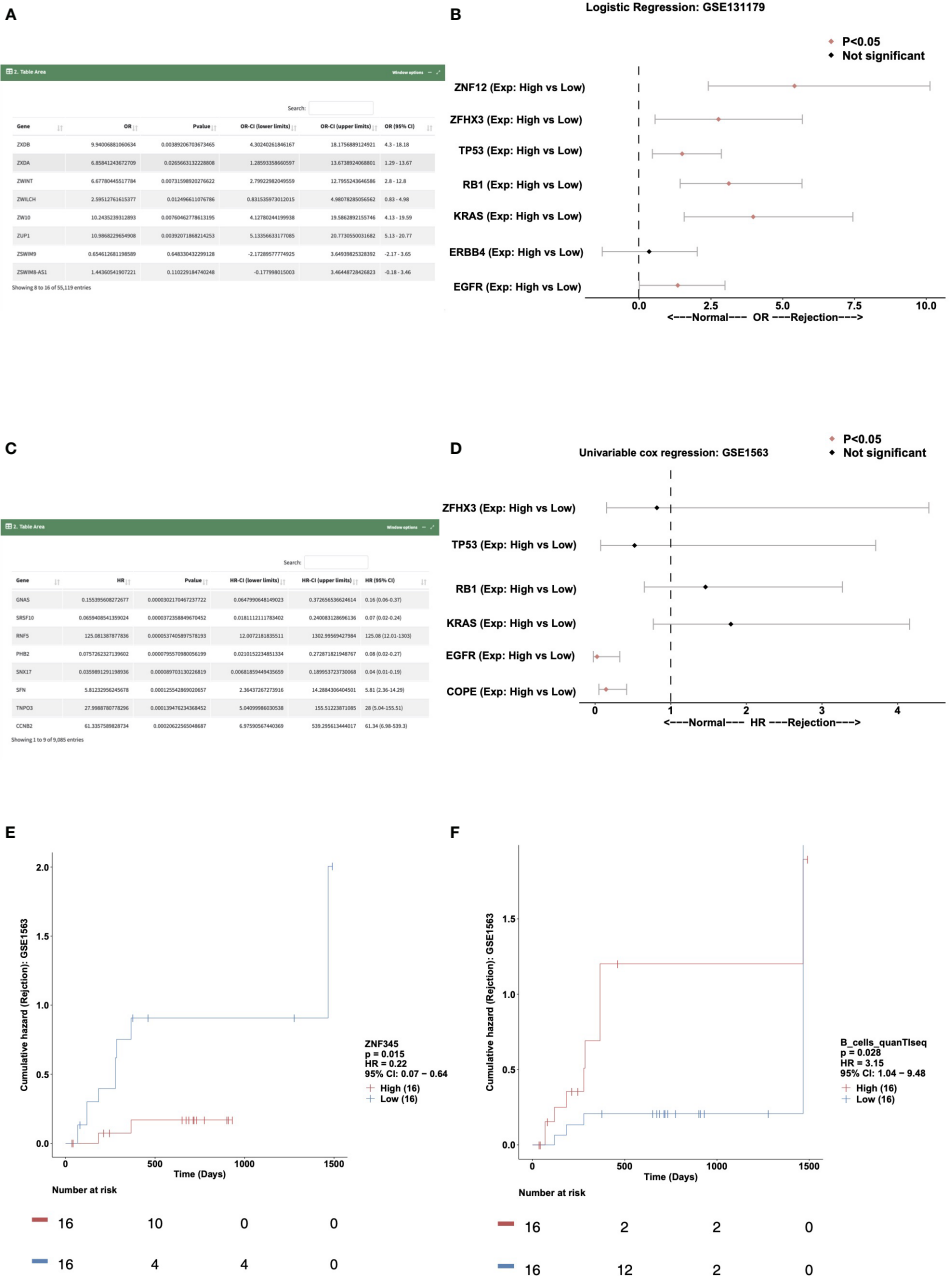
Example of the “Prognosis Analysis” tab. **(A)** Data table showing the result of the logistics regression model in cohort GSE131179. **(B)** Forest plot showing the OR and P value of the gene expressions for ZNF12, ZFHX3, TP53, RB1, KRAS, ERBB4, and EGFR in the GSE131179 cohort. **(C)** Data table showing the result of the univariable cox regression model in cohort GSE131179. **(D)** Forest plot showing the HR and P value of the gene expressions for ZFHX3, TP53, RB1, KRAS, EGFR, and COPE in the GSE131179 cohort. **(E, F)**. Survival characteristics of the patients included in the database, including gene expressions **(E)** and immune cells scores **(F)**.

To perform logical regression analysis on the expression data and immune cell data in a certain cohort, users can select the “Logistics Regression” sub-tab. In “DataTable,” users can choose to analyze different cohort samples and view the logistics regression results of genes and outcome variables. An example using “Variable Type: Gene Expressions; Cohort: GSE131179” for the rejection and control outcome variable is shown in **Figure 3A**. In “Forest Plot,” users can perform logistics regression analysis on the expression levels of these genes in the rejection and control groups by selecting, for example, “Cohort: GSE131179; Variable Type: Gene Expressions; Variables (max:10): ZNF12, ZFHX3, TP53, RB1, KRAS, ERBB4, EGFR” (**Figure 3B**).

The “Univariable Cox regression” sub-tab can be selected to analyze the expression data and immune cell data in a cohort. In the “DataTable,” users can choose to analyze the univariate Cox expression results between genes and the time of renal transplantation rejection in different cohort samples. An example using the criteria “Variable Type: gene expressions; Cohort: gse1563” is shown in **Figure 3C**. In “Forest Plot,” users can select “Cohort: GSE1563; Variable Type: gene expressions; Variables (Max: 10): ZFHX3, TP53, RB1, KRAS, EGFR, COPE” to analyze the expression levels of these genes and the univariable Cox regression analysis (**Figure 3D**).

Users can select the “Kaplan Meier” sub-tab to perform Kaplan Meier (KM) analysis on the expression data and immune cell data in a certain cohort. In “Gene-KM,” users can choose to analyze the relationship between the expression of a specific gene in different cohort samples and the time of renal transplantation rejection (e.g., “Cohort: GSE1563; Gene: ZNF345” shown in **Figure 3E**). In “Immune Cells-KM,” the user can explore the relationship between immune cell score and the time of renal transplantation rejection. An example using the criteria “Cohort: GSE1563; Immune cells: B_cells_quanTIseq” is shown in **Figure 3F**.

### Correlation analysis

**Figure 4:**
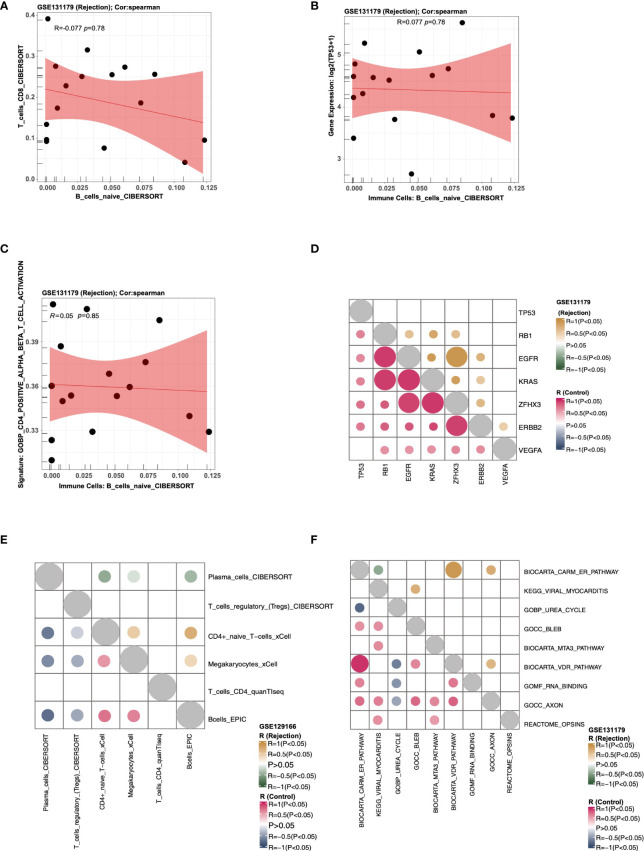
An example of the “Correlation Analysis” tab. **(A)** The correlation between the score of T_cells_CD8_CIBERSORT and the score of T_cells_CD8_CIBERSORT in cohort GSE131179 (Rejection samples). **(B)** The correlation between the score of B_cells_naive_CIBERSORT and the expression of TP53 in cohort GSE131179 (Rejection samples). **(C)** The correlation between the score of B_cells_naive_CIBERSORT and the score of GOBP_CD4_POSITIVE_ALPHA_BETA_T_CELL_ACTIVATION in cohort GSE131179 (Rejection samples). **(D)** Heatmap showing the correlation R value and P value among genes TP53, RB1, EGFR, KRAS, ZFHX3, ERBB2, and VEGFA) in the rejection and control groups of the GSE131179 cohort, respectively. **(E)** Heatmap showing the correlation R value and P value among the immune cells Plasma_cells_CIBERSORT, T_cells_regulatory_(Tregs)_CIBERSORT, CD4+_naive_T−cells_xCell, Megakaryocytes_xCell, T_cells_CD4_quanTIseq, and Bcells_EPIC in the rejection and control groups of the GSE131179 cohort, respectively. **(F)** Heatmap showing the correlation R value and P value among ssGSEA Pathways BIOCARTA_CARM_ER_PATHWAY, KEGG_VIRAL_MYOCARDITIS, GOBP_UREA_CYCLE, GOCC_BLEB, BIOCARTA_MTA3_PATHWAY, BIOCARTA_VDR_PATHWAY, GOMF_RNA_BINDING, GOCC_AXON, and REACTOME_OPSINS in the rejection and control groups of the GSE131179 cohort, respectively.

Users can select the “Scatter Plot” sub-tab to analyze the correlations among the expression data, immune cells, and pathway scores in a cohort, displaying them in a scatter plot. In “Immune Cells,” the user can, for example, select “Cohort: GSE131179; Immune cells: T_cells_CD8_CIBERSORT, B_cells_naive_CIBERSORT; Method for computing correlation coefficient: spearman; Samples Origin: Rejection” to analyze the correlation (R value and P value) between the T_cells_CD8_CIBERSORT and B_cells_naive_CIBERSORT in the rejection samples (GSE131179) (**Figure 4A**). In “Immune Cells-Genes,” users can select “Cohort: GSE131179; Immune cells: B_cells_naive_CIBERSORT; Genes: TP53; Method for computing correlation coefficient: spearman; Samples Origin: Rejection” to analyze the correlation coefficient and P value between B_cells_naive_CIBERSORT and TP53 expression levels (**Figure 4B**). In “Immune Cells-Pathways,” users can select “Cohort: GSE131179; Immune cells: B_cells_naive_CIBERSORT; Pathways: GOBP_CD4_POSITIVE_ALPHA_BETA_T_CELL_ACTIVATION; Method for computing correlation coefficient: spearman; Samples Origin: Rejection” to analyze the correlation coefficient and P value between the B_cells_naive_CIBERSORT and GOBP_CD4_POSITIVE_ALPHA_BETA_T_CELL_ACTIVATION in the rejection sample (GSE131179) (**Figure 4C**).

Using the “Heatmap” sub-tab, users can analyze the correlations among the expression data, immune cells, and pathway scores in a certain cohort, and display them as a heat map. In “Genes,” users can, for example, select “Cohort: GSE131179; Genes: (3~10): TP53, RB1, EGFR, KRAS, ZFHX3, ERBB2, VEGFA; Method for computing correlation coefficient: spearman” to analyze the correlation score and P value of the selected genes in the rejection and control groups, respectively (**Figure 4D**). In “Immune cells,” the user can choose to calculate the correlation coefficient and P values of selected immune cells from a specific cohort in the rejection and control groups. An example using the criteria “Cohort: GSE131179; Immune Cells: (3~10): Plasma_cells_CIBERSORT, T_cells_regulatory_(Tregs)_CIBERSORT, CD4+_naive_T−cells_xCell, Megakaryocytes_xCell, T_cells_CD4_quanTIseq, Bcells_EPIC; Method for computing correlation coefficient: spearman” is shown in **Figure 4E**. In “Pathways,” users can analyze the correlation coefficient and P values of selected signal pathway scores in the rejection and control groups respectively from a specific cohort. An example using the criteria “Cohort: GSE131179; Pathways: (3~10): BIOCARTA_CARM_ER_PATHWAY, KEGG_VIRAL_MYOCARDITIS, GOBP_UREA_CYCLE, GOCC_BLEB, BIOCARTA_MTA3_PATHWAY, BIOCARTA_VDR_PATHWAY, GOMF_RNA_BINDING, GOCC_AXON, REACTOME_OPSINS; Method for computing correlation coefficient: spearman” is shown in **Figure 4F**.

### Enrichment analysis

**Figure 5:**
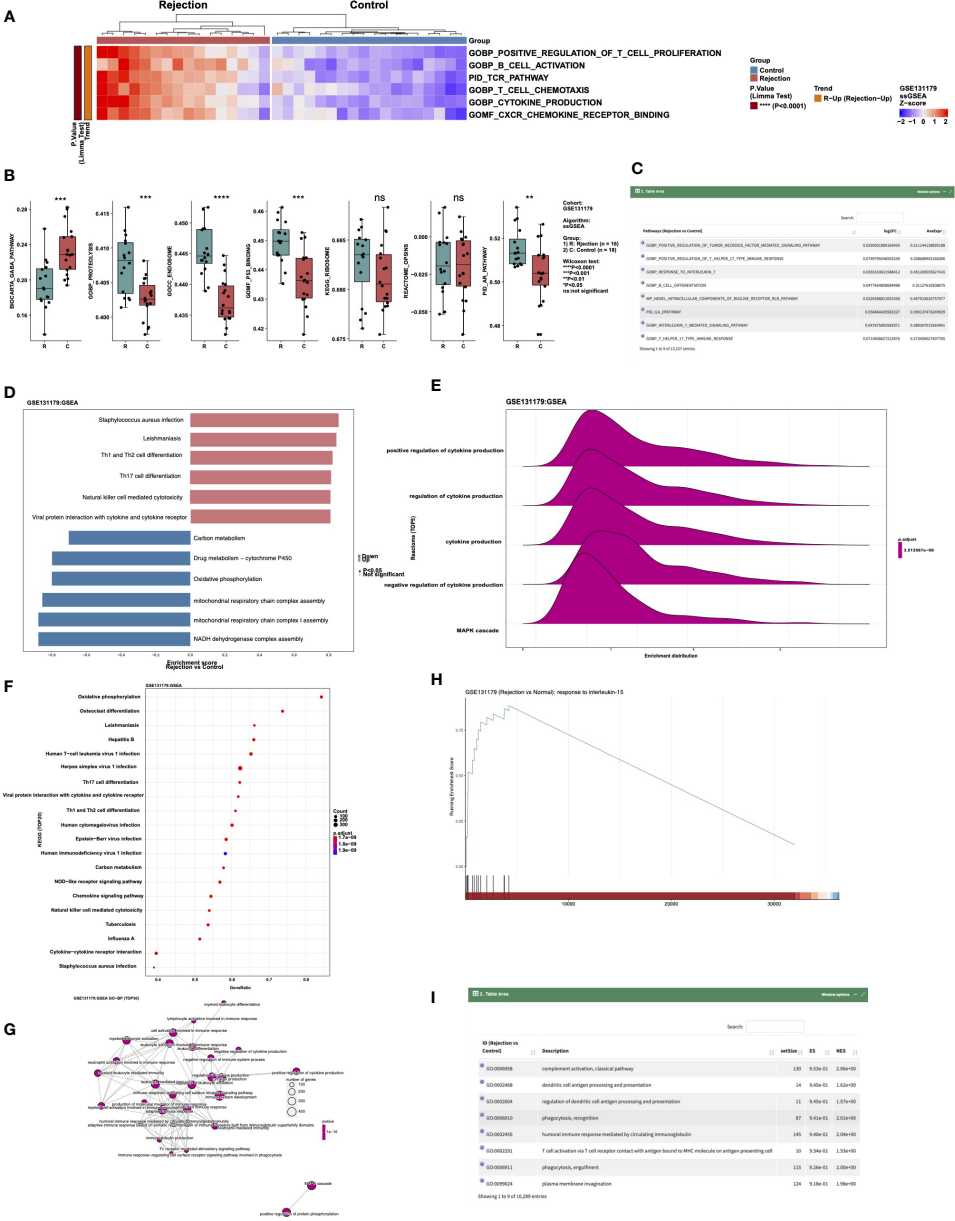
An example of the “Enrichment Analysis” tab. **(A)** Heatmap showing the differences in the scores of ssGSEA Pathways (GOBP_POSITIVE_REGULATION_OF_T_CELL_PROLIFERATION, GOBP_B_CELL_ACTIVATION. PID_TCR_PATHWAY GOBP_T_CELL_CHEMOTAXIS, GOBP_CYTOKINE_PRODUCTION and GOMF_CXCR_CHEMOKINE_RECEPTOR_BINDING) between rejection and control groups in cohort GSE131179. **(B)** Boxplot showing the difference in the scores of ssGSEA Pathways BIOCARTA_GABA_PATHWAY, GOBP_PROTEOLYSIS, GOCC_ENDOSOME, GOMF_P53_BINDING, KEGG_RIBOSOME, REACTOME_OPSINS, and PID_AR_PATHWAY between rejection and control groups in the GSE131179 cohort. **(C)** Data table showing the differences in the scores of the ssGSEA Pathways between rejection and control groups in the GSE131179 cohort. **(D–H)** Barplot **(D)**, ridgeline plot **(E)**, dot plot **(F)**, emap plot **(G)**, GSEA plot **(H)** and data table **(I)** of the GSEA result in cohort GSE131179.

To analyze the difference between ssGSEA scores in a cohort, users can select the “ssGSEA” sub-tab. In “Heatmap,” users can, for example, select “Cohort: GSE131179; Search, select 5~20 pathways:: GOBP_POSITIVE_REGULATION_OF_T_CELL_PROLIFERATION, GOBP_B_CELL_ACTIVATION. PID_TCR_PATHWAY GOBP_T_CELL_CHEMOTAXIS, GOBP_CYTOKINE_PRODUCTION, GOMF_CXCR_CHEMOKINE_RECEPTOR_BINDING; Cluster Method: ward.D “ to analyze the these signaling pathways of GSE131179 in which ssGSEA scores are significantly increased or decreased (**Figure 5A**). In “Boxplot,” the user can perform a Wilcox test to compare the differences in selected channel scores between the rejection and control groups. An example using the criteria “Cohort: GSE131179; Pathways (max:10): BIOCARTA_GABA_PATHWAY, GOBP_PROTEOLYSIS, GOCC_ENDOSOME, GOMF_P53_BINDING, KEGG_RIBOSOME, REACTOME_OPSINS, PID_AR_PATHWAY” can be seen in **Figure 5B**. The difference analysis results of ssGSEA scores between the rejection and control groups are shown in **Figure 5C**.

Users can display the GSEA results in a certain cohort by selecting the “GSEA” sub tab. In “Barplot,” users can, for example, select “Cohort: GSE131179; Analysis Type: 1.Top-5&Down-5 Pathways” to show the enrichment scores (ES) of the five channels that are significantly up- and down-regulated after enrichment analysis in this data set (**Figure 5D**). In “Ridgeline Plot,” users can select “Cohort: GSE131179; Pathway Collection: Reactome; Analysis Type: Advanced: Choose Top-N Pathways; Top-N Pathways: N = 5” to show the ridgeline plot of the top 5 Reactome signaling pathways in this dataset (**Figure 5E**). In “Dot Plot,” users can select “Cohort: GSE131179; Pathway Collection: KEGG; Analysis Type: Advanced: Choose Top-N Pathways; Top-N Pathways: N = 20” to show the dot plot of the top 20 KEGG signaling pathways in this dataset (**Figure 5F**). In “Emap Plot”, users can select “Pathway Collection: GO-BP; Analysis Type: Default: Top-30 Pathways” to show the emap plot (**Figure 5G**). In “GSEA,” users can select “Cohort: GSE131179; Pathway Collection: GO-BP; Please choose a pathway: response to interleukin-15” to show the GSEA plot (**Figure 5H**). A display of the analysis results of GSEA from different data sets can be seen in **Figure 5I**.

### ROC analysis

**Figure 6:**
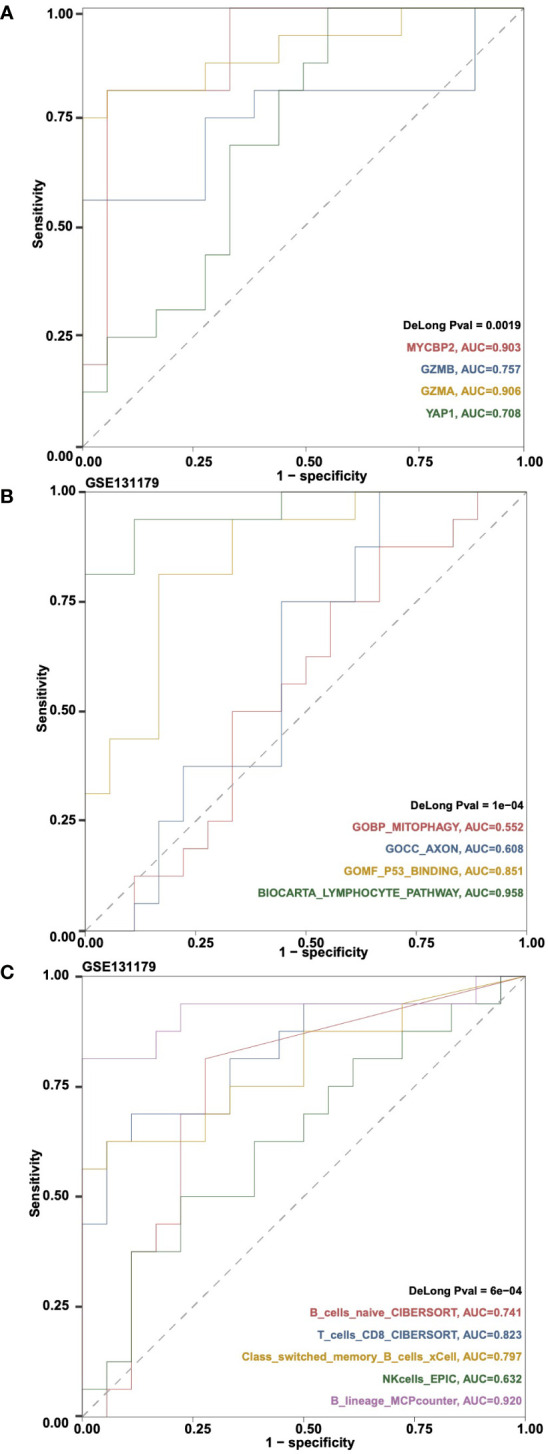
An example of the “ROC Analysis” tab. **(A)** ROC analysis results on the gene expressions MYCBP2, GZMB, GZMA and YAP1 in cohortGSE131179. **(B)** ROC analysis results on the ssGSEA pathways GOBP_MITOPHAGY, GOCC_AXON, GOMF_P53_BINDING and BIOCARTA_LYMPHOCYTE_PATHWAY in cohort GSE131179. **(C)** ROC analysis results on the immune cells B_cells_naive_CIBERSORT, T_cells_CD8_CIBERSORT, Class-switched_memory_B-cells_xCell, NKcells_EPIC, and B_lineage_MCPcounter in cohort GSE131179.

The “Gene Expressions” sub-tab can be used to perform ROC analysis and calculate the Area Under Curve (AUC) of gene expression and rejection and control variables in a certain cohort. For example, users can select “Cohort: GSE131179; Gene Symbol (max:5): MYCBP2, GZMB, GZMA, YAP1” to perform ROC analysis on the selected genes (**Figure 6A**). Users can select the “ssGSEA Pathways” sub-tab to perform ROC analysis on the ssGSEA score of the rejection and control groups in a certain cohort and calculate AUC. For example, users can select “Cohort: GSE131179; Pathways (max:5): GOBP_MITOPHAGY, GOCC_AXON, GOMF_P53_BINDING, BIOCARTA_LYMPHOCYTE_PATHWAY” to perform ROC analysis on the selected ssGSEA signature scores (**Figure 6B**). In addition, users can select the “Immune cells” sub tab to perform ROC analysis on immune cells based on the rejection and control variables in a certain cohort and calculate AUC. An example using the criteria “Cohort: GSE131179; Immune Cells (max:5): B_cells_naive_CIBERSORT, T_cells_CD8_CIBERSORT, Class-switched_memory_B-cells_xCell, NKcells_EPIC, B_lineage_MCPcounter” is shown in **Figure 6C**.

### Datasets and FAQ

In the “Datasets” tab, users can view all data set information related to CARARIME, including DataSet Name, SampleNumber, Species, SampleType, ExperimentType, Summary, Overall Design, and Platforms. Additionally, the differences of the clinical characteristics between the rejection and control group were detailed in the **Table S2**. In the FAQ tab, users can view answers to common questions regarding CARARIME.

### Data integration analysis and validation

**Figure 7:**
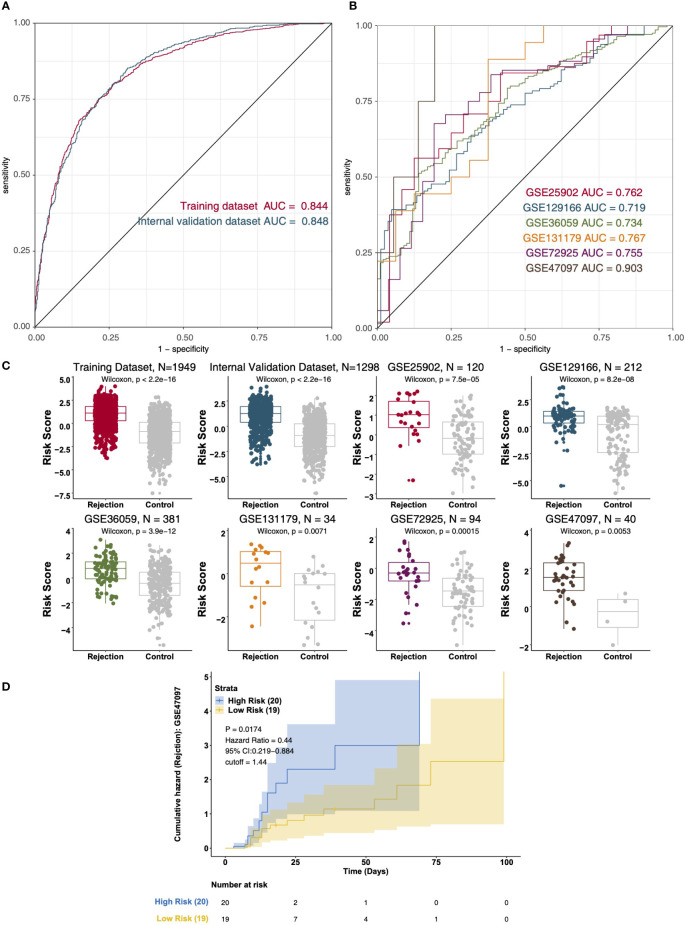
The result of the data integration Analysis. **(A)** ROC analysis of risk scores based on the training data dataset and internal validation dataset. **(B)** ROC analysis of risk scores based on the external validation dataset (GSE25902, GSE129166, GSE36059, GSE131179, GSE72925, and GSE47097). **(C)** Boxplot showing the difference in the risk scores between rejection and control groups in the training dataset, internal validation dataset, and external validation dataset (GSE25902, GSE129166, GSE36059, GSE131179, GSE72925, and GSE47097). Survival characteristics of the patients in the GSE47097 based on the risk scores **(D)**.

Based on the multivariable logistic regression model, we found that CD4+ T_cells_CD4_memory_resting, T_cells_regulatory_Tregs, Macrophages_M1, and Mast_cells_resting could effectively predict the renal transplant rejection status of the KTRs (**Table S3**). The ROC curve indicated the well-predicted ability of the risk score model in the training dataset [Area under the ROC Curve (AUC) = 0.844] and the internal validation dataset [AUC = 0.848] (**Figure 7A**) and external validation dataset (**Figure 7B**), respectively. Additionally, the risk score of the rejection group is significantly higher than that of the control group in both the internal validation dataset and the external validation dataset (**Figure 7C**). We further divided the KTRs of the GSE47097 into a high-risk group (high risk) and a low-risk group (low risk) based on the median risk score. Moreover, we compared the cumulative morbidity of the two groups. The cumulative morbidity rate was significantly higher in the high-risk group compared with the low risk score group in the GSE47097 [P = 0.0174, Hazard Ratio (HR) = 0.44, 95% CI: 0.219-0.884, **Figure 7D**].

## Discussion

As an interactive web tool, CARARIME uses data sets from rejection and non-rejection post renal transplantation samples, including expression data, data on rejection status, and data on the time of rejection. This web tool provides researchers who are not experienced in computer programming with the possibility of exploring the immune microenvironment in samples from patients with renal transplant rejection. Users can easily study the gene expression of the rejection and control groups in different renal transplantation data sets by clicking the appropriate options with the mouse. In addition, prognosis analysis also provides users with an opportunity to study the relationship between gene expression, immune cell content, signal pathway activation score, state after transplantation, and rejection time after transplantation. In recent years, several studies have shown that the immune microenvironment plays a vital role in the rejection of renal transplants. This includes immune cells (T cells, CTLs, NKs, and Tregs) and inflammatory mediators (IFN-γ, granzyme, perforin, TNF-α, and IL-17) (2, 3, 8). However, at present, the specific mechanism of how the immune microenvironment affects the occurrence and development of renal transplant rejection is not fully understood. Therefore, it is important to explore the role of the immune microenvironment in renal transplant rejection, and one way to do this is *via* bioinformatics and the relevant published data. In CARARIME, we have developed a simple and convenient web tool that does this, providing a convenient way for researchers and clinicians to study the immune microenvironment in the context of renal transplantation rejection.

## Conclusions

In this study, CARARIME can help researchers easily analyze the immune microenvironment in the context of renal transplant rejection by clicking on the available options. CARARIME can be found in http://www.cararime.com.

## Data availability statement

The datasets presented in this study can be found in online repositories. The names of the repository/repositories and accession number(s) can be found in the article/**Supplementary Material**.

## Author contributions

Conceptualization, MZ, YoL: Formal analysis. DL, XL: Software. DL, SZ, XL: Supervision. MZ, YoL: Resources. MZ, YoL, DL: Visualization. DL, SZ, XL: Writing-original draft. DL, SZ, XL, WJ, JZ, JH, GL, JL, ZG, YuL, SY, SL, HC, YG, ML, LF, LL: Writing-review & editing. DL, SZ, XL, WJ, JZ, JH, GL, JL, ZG, YuL, SY, SL, HC, YG, ML, LF, LL, MZ, YoL. All authors contributed to the article and approved the submitted version.

## Funding

This work was supported by Basic and Applied Basic Research Foundation of Guangdong Province (Grant No. 2022A1515012304), and the National Natural Science Foundation of China (Grant No. 82170764).

## Conflict of interest

The authors declare that the research was conducted in the absence of any commercial or financial relationships that could be construed as a potential conflict of interest.

## Publisher’s note

All claims expressed in this article are solely those of the authors and do not necessarily represent those of their affiliated organizations, or those of the publisher, the editors and the reviewers. Any product that may be evaluated in this article, or claim that may be made by its manufacturer, is not guaranteed or endorsed by the publisher.

## Supplementary material

Supplementary Table 1 The details of the clinical cohorts used in this study.

Supplementary Table 2 The differences of the clinical characteristics between rejection and control groups.

Supplementary Table 3 The result of the multivariable logistic regression model in the training dataset.

## Abbreviations

CARARIME, Comprehensive Analysis of Renal Allograft Rerejction in Immune Microenvironment; GSEA, Gene Set Enrichment Anaysis; ssGSEA, Single Sample Gene Set Enrichment Anaysis; APCs, anti-presenting cells; NKs, natural killer cells; CTLs, cytotoxic T lymphocytes; Th17, T helper 17; MIP, macrophage inflammatory protein; GEO, Gene Expression Omnibus; CHIP, chromatin immunoprecipitation; Tregs, T cells regulatory; KM, Kaplan Meier; ES, enrichment scores; AUC, Area Under Curve.
